# Combining ability for agronomic traits among commercial maize hybrids under low and high nitrogen inputs targeting the development of breeding populations

**DOI:** 10.1371/journal.pone.0309296

**Published:** 2024-10-07

**Authors:** Luiz Silva Luz, Helber Moreira dos Reis, Noé Mitterhofer Eiterer Ponce de Leon da Costa, Flaviane Ribeiro Carvalho, Diego Gonçalves Caixeta, Rodrigo Oliveira DeLima

**Affiliations:** Department of Agronomy, Universidade Federal de Viçosa, Viçosa, Minas Gerais, Brazil; Osmania University, INDIA

## Abstract

Commercial hybrids are the main germplasm source for developing maize lines in breeding programs in Brazil; additionally, nitrogen (N) is one the major limiting maize production in Brazilian tropical areas. Here, we assessed the combining ability among ten commercial hybrids under contrasting N inputs and selected the best parental hybrids to develop breeding populations for optimal and N-stress environments. We evaluated the 45 F_1_ crosses for agronomic traits under contrasting N inputs and over two summer seasons. A mixed model approach was used to estimate the variance components of general combining ability (GCA) and specific combining ability (SCA) as well as to predict the GCA and SCA effects. N-stress caused a reduction in GY (33.25%) of F_1_ crosses averaged across seasons. We found presence of combining ability (GCA and SCA) x N input interaction for grain yield (GY), days to pollen and plant stature. The parental hybrids showed differences in GCA for cycle and plant stature but not for GY, irrespective of N inputs. Additionally, the variance components of SCA were not significant (P>0.10) for GY under LN, whereas SCA was the major component accounting for variation among F_1_ crosses under HN. Based on estimates of GCA effects for cycle and plant height, we selected the hybrids BAL188, BM3061, GNZ7210, BRS1060 and DKB390 as sources of favorable alleles for earlier maturing and shorter stature maize for both N conditions and suggested that hybrids GNZ7201 and DKB390, and AG1051 and NS70, which presented very small estimates of SCA for GY, must be recombined to develop two synthetic populations to begin a reciprocal recurrent selection program, mainly for non N-stress environments.

## Introduction

Currently, Brazilian maize production (around 100 million metric tons) represents approximately 10% of global maize production [1]. However, Brazilian maize yield is still low (5.72 Mg ha^-1^) compared with that of the United States (11.07 Mg ha^-1^) and Argentina (8.36 Mg ha^-1^), where maize is produced in temperate and/or subtropical environments. In Brazil, over 75% of maize is harvested under tropical areas, where maize production is greatly constrained by adverse environmental conditions, such as high nighttime temperatures, drought occurrence and the predominance of poor soils with mineral deficiencies, especially for low N (LN) and phosphorus [2–4]. Low N stress, along with drought, is the major cause of maize yield loss across tropical environments [5–7].

Nitrogen is the nutrient required in the largest amount for maize production and is considered the second most limiting factor, after water, for maize growth and development [5, 6, 8]. In the last years, Brazil applied approximately 12 Mg de N fertilizer per year in its agriculture, and 75% (around 9 Mg) of this amount were imported from other countries, mainly from Russia and China [9]. Moreover, N-use efficiency in maize is estimated to be far less than 50% [10, 11], and thereby up to 50% of applied N is lost to the environment by leaching, denitrification, and volatilization; this predominantly occurs in tropical environments, where N cycling is faster and soil organic matter is lower compared with that of temperate soils [2, 12, 13]. Thus, a way to Brazil become less dependent of the importation of N fertilizer from other countries, decrease the high N rates in fertilizers applied by maize growers, reduce N losses to environmental, and also increase maize grain yield under LN stress is to develop maize hybrids with improved N-use efficiency by using breeding methods [7, 11, 14]. Hence, public and private maize breeding programs have intensified their efforts in genetic improvement for LN stress and drought tolerance, mainly across Brazilian and African tropical environments [4, 7, 15, 16]. In a recent study, [17] assessed the field performance of 114 maize hybrids currently cropped in Brazil and found large genotypic variation for grain yield and N-use traits among hybrids even under LN stress conditions. However, maize hybrids responded differently to N inputs, and the authors suggested that LN stress environments must be included in the pipeline of maize breeding programs that target high-yielding and N-use efficient hybrids.

High-yielding and N-use efficient maize hybrids for LN stress environments could be obtained through the accumulation of favorable alleles for low N tolerance in both parental lines [18–20]. According to [21], the development of inbred lines under LN resulted in maize hybrids that are better adapted to LN stress than hybrids derived from inbred lines improved across optimal N conditions. Therefore, the development of maize breeding populations and inbred lines under contrasting N inputs might be an important strategy to develop modern hybrids with improved N-use efficiency and high yielding under LN environments, mainly in tropical areas. In Brazil, commercial hybrids are the main germplasm source for developing new inbred lines in most maize breeding programs [22–24]. Commercial hybrids are developed from crossing among elite inbred lines and, consequently, breeding populations developed from selfing of commercial hybrids have a high frequency of favorable alleles for many agronomic traits [25]. Also, those populations have a high frequency of alleles “adapted” to diverse environments since commercial are extensively exposure to high selection pressure by maize growers, mainly under Brazilian tropical environments. Thus, many studies have reported the genetic potential of breeding populations derived from commercial hybrids to develop new hybrid lines and hybrids [26–30]. However, all these studies were conducted under non-N stress conditions, and there is no information regarding the selection of commercial hybrids that target the development of breeding populations for LN environments.

The combining ability among genotypes has been widely adopted in maize breeding programs to develop and select breeding populations, compare the performance of inbred lines in hybrid combinations, and develop high heterotic and yielding hybrids [31–34]. Large differences among commercial hybrids in general (GCA) and specific (SCA) combining ability have been reported for agronomic traits in maize, mainly under non-N stress environments [27–30]. Then, promising commercial hybrids were selected based on GCA effects for use either as breeding populations and/or sources of new inbred lines. Additionally, commercial hybrids that present favorable alleles for a target trait (large GCA effects) and low genetic divergence among them (negative or small SCA effects) have been recombined to develop synthetic populations [29, 33, 35, 36]. These have been improved through intrapopulation recurrent selection and/or used as base populations in a reciprocal recurrent selection (RSS) program targeting the development of superior diverse inbred lines [24, 27, 37–40]. Interestingly, there is no study assessing the combining ability among commercial hybrids across low N-stress environments.

Considering the above, we hypothesized that commercial maize hybrids differ in supplying favorable alleles with additive effects to their progenies and also there is a strong allelic complementation among them under LN stress and optimal conditions. Our objectives were to i) estimate the combining ability for agronomic traits among maize commercial hybrids under contrasting N inputs and ii) identify maize commercial hybrids with desirable combining ability to be used as germplasm sources in the development of new inbred lines for optimum and N-stress conditions.

## Materials and methods

### Plant material

Ten maize commercial hybrids were selected for our study based on their outstanding performance for grain yield and N-use efficiency under contrasting N inputs across tropical environments [17]. Moreover, they have modern plant architecture and showed moderate-to-high tolerance to LN stress across tropical environments. They were developed by ten different seed companies and were non-transgenic (conventional) hybrids (Table 1). The ten hybrids were crossed using a diallel mating design according to Griffing’s method 4 [41]. Crosses were made during the 2017 winter season, and each hybrid was used as both male and female. Seeds of reciprocal crosses were bulked to form 45 F_1_ crosses.

**Table 1:** Description of the ten tropical maize commercial hybrids used as parents in this study.

Hybrids	Type	Kernel texture	Cycle	Seed Company
AG1051	Double cross	Dent	Semi-early	Agroceres
BAL188	Three-way cross	Semi Flint	Very early	Balu
BG7049	Three-way cross	Semi Flint	Early	DuPont
BM3061	Three-way cross	Dent	Early	Biomatrix
BRS1060	Single cross	Semi Dent	Semi-early	Embrapa
DKB390	Single cross	Semi Flint	Early	Dekalb
GNZ7201	Single cross	Semi Dent	Early	Geneze
NS70	Single cross	Semi Flint	Early	Nidera
SHS4070	Single cross	Dent	Semi-early	Santa Helena
XB8018	Double cross	Flint	Early	Semeali

### Trial management and experimental design

Field experiments were carried out at the Experimental Station of the Federal University of Viçosa, located in Coimbra (lat. 20°49’46.5”S; long. 42°45’51.1”W; alt. 715 m a.s.l.), Minas Gerais State, Brazil, during the 2017/2018 and 2018/2019 summer seasons. During both growing seasons, the experiments were sowed under no-tillage conditions. The soil type was a dystrophic Red‒Yellow Argisol. The soils were sampled from 0 to 20 cm depth in the fall before planting the plots. The soil samples were analyzed for pH, organic matter, and available P, K, Mg, Ca, and Al (Table 2). According to the soil analysis, 100 kg ha^-1^ P_2_O_5_, 60 kg ha^-1^ K_2_O, and 20 kg ha^-1^ N were applied before sowing.

**Table 2:** Chemical properties of the soils in each field experiment.

N inputs	pH	H+Al	Al	Ca	Mg	K	P	Organic matter
		cmolc dm^-3^				mg dm^-3^		dag kg^-1^
HN	5.2	3.80	0.0	2.4	1.3	135	26.1	3.35
LN	5.4	3.80	0.0	2.5	1.2	101	21.3	1.98

**Fig 1:**
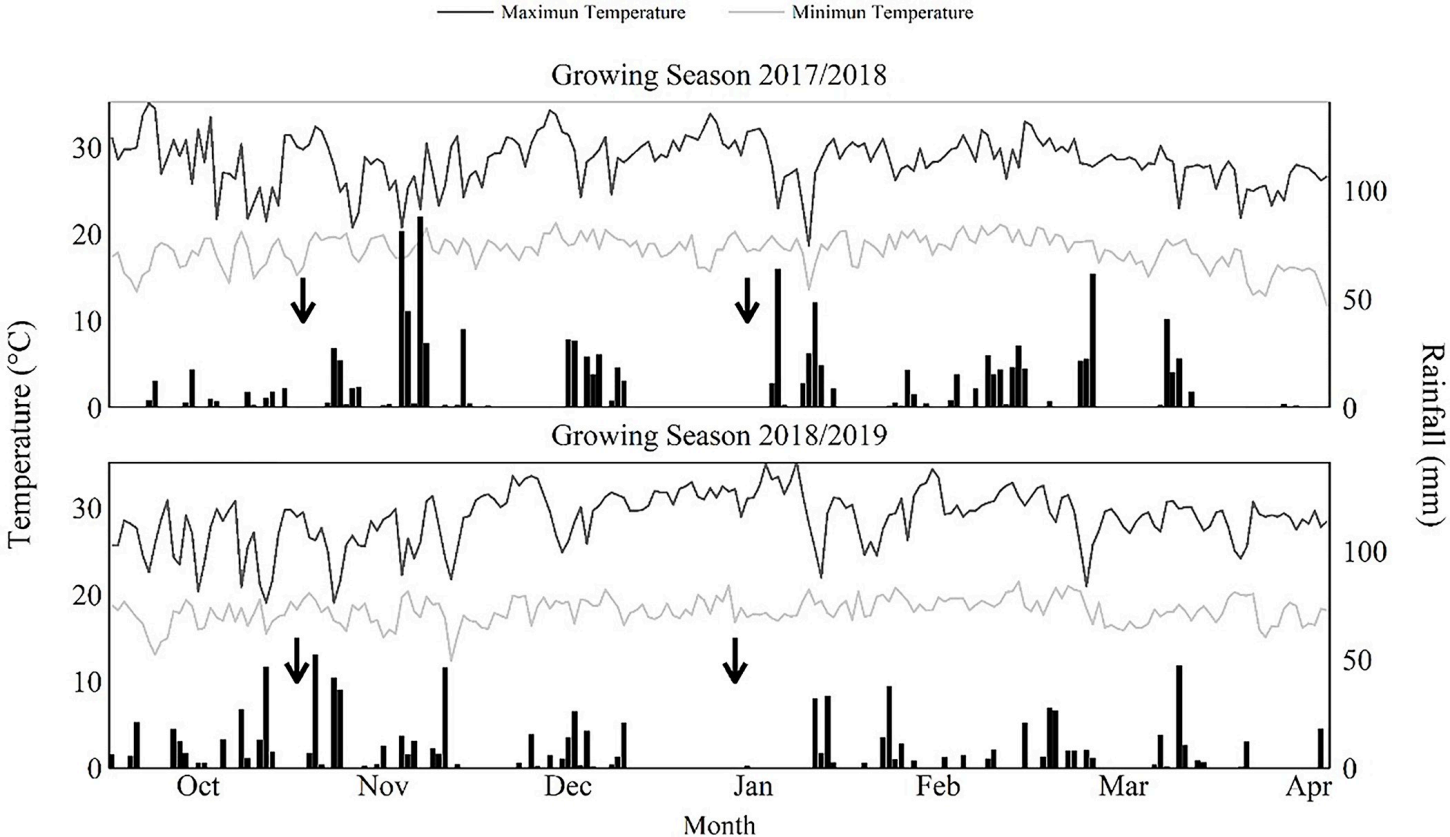
Daily minimum temperature, maximum temperature, and rainfall during the 2017/2018 and 2018/2019 maize growing seasons. Coimbra, MG, Brazil. From left to right, the arrows points to the date for sowing November 15, 2017, and November 14, 2018, and for R1 stage. Source: Data from INMET.

In both seasons, the 45 F_1_ crosses were evaluated under two contrasting N inputs: an LN corresponding to 20 kg ha^-1^ of N, applied before sowing, and an HN corresponding to 200 kg ha^-1^ of N: 20 kg ha^-1^ of N, applied before sowing, and 180 kg ha^-1^ of N that were broadcast-applied as dry urea (43-00-00) after plant emergence. Urea was split into two equal amounts and applied to all hybrids around the V3 and V6 growth stages [42]. Experiments under LN were planted in fields that had been depleted for N by growing unfertilized non-leguminous crops for several years and removing the biomass after each season. The experiments were conducted in rain-fed conditions with no irrigation being applied (Fig 1). In all experiments, the weeds were controlled with pre-emergence herbicide followed by post-emergence herbicide applications using standard agronomic practices. Other trial management was the same for all experiments. All maize seeds were treated in a similar manner with CropStar^®^ (imidacloprid, thiocarb) and Maxim Advanced^®^ (metalaxyl-M, thiabendazole, fludioxonil).

The LN and HN experiments were grown side-by-side on the same field, and each trial (L × N input combination) was laid out in an alpha-lattice incomplete block design with three replications. The plots were 5 m long, with two rows spaced 80 cm apart and a plot size of 8.0 m^2^. Seeds were sown on November 15, 2017, and November 14, 2018, in the 2017/2018 and 2018/2019 growing seasons, respectively. Plots were overseeded with hand planters, then thinned at the V4 stage to a plant population of 62,500 plants ha^-1^, which is the optimum population for maize hybrids growing in those environments.

### Trait measurements

We evaluated five agronomic traits, namely, days to pollen shedding (DTP), days to silking (DTS), plant height (PH), ear height (EH), and grain yield (GY). Days to pollen shedding and DTS were recorded as the number of days from sowing to the day when 50% of anthers extruded outside the glumes and when silk became visible, respectively; PH and EH were measured in centimeters as the distance from the ground level to the collar of the uppermost leaf and to the collar of the upper ear leaf, respectively; and GY was recorded for all the ears in the plots at physiological maturity. The ears were shelled, the grain weight and grain moisture percentage were recorded, and the GY (kg ha^-1^) was calculated at 145 g kg^-1^ moisture.

### Statistical analysis

Variance components were estimated using the restricted maximum likelihood (REML) approach [43], and the GCA and SCA effects were predicted using the best linear unbiased predictors (BLUP) approach [44]. Thus, a mixed model implemented in the R package “ASReml" [45] was used to estimate the variance components of general combining ability (GCA) and specific combining ability (SCA) and to predict the GCA and SCA effects according to the diallel model described in Griffing’s method 4 [41]. Then, the phenotypic values were modeled according Eq 1 (analysis by N input and across seasons) and Eq 2 (combined analysis across N inputs and seasons):

$$y=X\tau +Z_{g}g+Z_{s}s+Z_{g_{Y}}g_{Y}+Z_{s_{Y}}s_{Y}+Z_{b_{Y}}b_{Y}+e,$$ (1)

and

$$y=X\tau +Z_{g}g+Z_{s}s+Z_{g_{T}}g_{T}+Z_{s_{T}}s_{T}+Z_{b_{T}}b_{T}+e,$$ (2)

where $Z_{g_{T}}g_{T}=Z_{g_{Y}}g_{Y}+Z_{g_{N}}g_{N}+Z_{g_{YxN}}g_{YxN}$ and $Z_{s_{T}}S_{T}=Z_{s_{Y}}s_{Y}+Z_{s_{N}}s_{N}+Z_{s_{YxN}}S_{YxN}$; each effect was defined as follows: y is the vector of phenotypic values; τ is the vector of fixed effects divided into replications and means of F_1_ crosses; g is the vector of random GCA effects, with g ~ N(0, $\sigma_{g}^{2}I_{g}$); s is the vector of random SCA effects, with s ~ N(0,$\sigma_{s}^{2}I_{s}$); g_Y_ is the vector of random effects of GCA x year interaction, with g_Y_ ~ N(0,$\sigma_{g_{Y}}^{2}I_{g_{Y}}$); s_Y_ is the vector of random effects of SCA x year interaction, with s_Y_ ~ N(0,$\sigma_{s_{Y}}^{2}I_{s_{Y}}$); g_N_ is the vector of random effects of GCA x N input interaction, with g_N_ ~ N(0,$\sigma_{g_{N}}^{2}I_{g_{N}}$); s_N_ is the vector of random effects of SCA x N input interaction, with s_N_ ~ N(0,$\sigma_{s_{N}}^{2}I_{s_{N}}$); g_YxN_ is the vector of random effects of GCA x N input x year interaction, with g_YxN_ ~ N(0,$\sigma_{g_{YxN}}^{2}I_{g_{YxN}}$); S_YxN_ is the vector of random effects of SCA x N input x year interaction, with s_YxN_ ~ N(0,$\sigma_{s_{YxN}}^{2}I_{s_{YxN}}$); b_Y_ is the vector of random effects of block within replication within year, with b_Y_ ~ N(0,$\sigma_{b_{Y}}^{2}I_{b_{Y}}$); b_T_ is the vector of random effects of block within replication within N input within year, with b_T_ ~ N(0,$\sigma_{b_{T}}^{2}I_{b_{T}}$), and e is a random error vector, with e ~ N(0, σ^2^I).$X,Z_{g},Z_{s},Z_{g_{Y}},Z_{s_{Y}},Z_{g_{N}},Z_{s_{N}},Z_{g_{YxN}},Z_{s_{YxN}},Z_{b_{Y}}$ and $Z_{b_{T}}$ are the design matrices of the random effects $g,s,g_{Y},s_{Y},g_{N},s_{N},g_{YxN},S_{YxN},b_{Y}$ and b_T_, respectively; and $I_{g},I_{s},I_{g_{Y}},I_{s_{Y}},I_{g_{N}},I_{s_{N}},I_{g_{YxN}},I_{s_{YxN}},b_{Y},I_{b_{T}}$ and I are the identity matrices associated with the (co)variance matrices of the random effects $g,s,g_{Y},s_{Y},g_{N},s_{N},g_{YxN},S_{YxN},b_{Y},b_{T}$ and e, respectively. As we employed mixed model approach to analyze the data, a likelihood ratio test (LRT) through deviance analysis was used to test the random effects via the chi-square statistic instead of F-test from analysis of variance [46]. First, we calculated the logarithm of maximum point of the likelihood function residual (L) to the reduced (without tested random effect) and full model (with all tested effects). Then, we obtained of deviance (D) as follows: D = −2*log (L) for both the full (D_f_) and reduced D_r_ models. After that, we obtained the likelihood ratio (LR) by the difference between the deviances of reduced and full model, as follows: LR = D_f_−D_r_. Finally, we tested, by LRT, the significance of the LR using the chi-square with a degree free.

## Results

### Weather conditions

Both seasons were characterized by optimum air temperatures during all maize growth phases (Fig 1). The average minimum temperature almost did not change between seasons in the experimental field, whereas the average maximum temperature had a slight range between seasons. Overall, the average maximum temperature was marginally higher during the 2018/2019 season than during the 2017/2018 season, mainly during the flowering stage. The rainfall distribution changed significantly between the growing seasons, with a larger and better distribution occurring during the 2017/2018 season (1,006.2 mm, from sowing to physiological maturity) than in the 2018/2019 season (643.3 mm). However, during both seasons, the rainfall amount reported in January 2018 (129.2 mm) and 2019 (29.8 mm) was lower than normal with lower precipitation occurring during the critical period for kernel number determination, i.e., 15 days bracketing silking (R1 ± 15 d).

### Low N effects on means and coefficient of variation

We observed that all measured traits were affected by N levels, mainly GY, which was highly affected by N stress among the tested traits (Table 3). Thus, the LN stress caused a reduction in the mean genotypic values of F_1_ crosses for PH (10.54%), EH (14.09%) and GY (33.25%) and an increase in their means for DTS (2.64%) and DTP (5.78%). The coefficient of variation (CV) was slightly higher under LN input than under HN input, and traits showed low to intermediate CV values. The values ranged from 2.16% (DTP) to 18.03% (GY) under LN and from 1.76% (DTP) to 14.58% (GY) under HN.

**Table 3:** Variance components estimates of general (${\hat{\sigma }}_{GCA}^{2}$) and specific (${\hat{\sigma }}_{SCA}^{2}$) combining ability, residual variance estimates (${\hat{\sigma }}^{2}$), mean, coefficient of variation (CV%) and percentage of reduction in the means for five agronomic traits measured in 45 F_1_ crosses evaluated under LH and HN input and over two summer seasons in Brazil.

Parameters	DTP^1/^ (days)	DTS (days)	PH (cm)	EH (cm)	GY (kg kg^-1^)
${\hat{\sigma }}_{GCA}^{2}$	0.71*^2/^	1.19*	31.84*	51.97*	0.16*
${\hat{\sigma }}_{SCA}^{2}$	0.45*	0.86*	15.94*	11.42*	294,134.50*
${\hat{\sigma }}_{GCAxN}^{2}$	2.17x10^-7^*	2.91x10^-7^	0.61*	0.05*	0.16*
${\hat{\sigma }}_{SCAxN}^{2}$	6.87x10^-8^*	7.71x10^-7^	1.59x10^-5^*	8.50x10^-6^*	113,598.20*
${\hat{\sigma }}_{GCAxY}^{2}$	0.05*	0.02*	1.59x10^-5^*	0.15*	73,127.80*
${\hat{\sigma }}_{SCAxY}^{2}$	2.17x10^-7^*	1.25x10^-6^*	1.59x10^-5^*	2.97x10^-5^*	26,450.01*
${\hat{\sigma }}_{GCAxNxY}^{2}$	0.26*	0.01	1.04x10^-5^*	0.00*	179,508.60*
${\hat{\sigma }}_{SCAxNxY}^{2}$	2.17x10^-7^*	2.91x10^-7^	9.08x10^-6^*	1.36x10^-5^*	0.16*
${\hat{\sigma }}^{2}$	2.14	2.87	41.21	97.64	1,597,630.00
Mean	70.05	70.77	208.08	109.74	6,969.90
CV%	2.09	2.39	3.09	9.00	18.14
% reduction of the mean^3/^	-5.75	-2.64	10.54	14.09	33.25

^1/^DTP: days to pollen shedding; DTS: days to silking; PH: plant height; EH: ear height, and GY: grain yield.

^2/^* Significant at 0.05 by the likelihood ratio test.

^3/^ Percentage of reduction in the means of tested traits in response to LN stress estimated for each trait as follows: {[(HN−LN)/HN]×100}.

### Variance components estimates of combining ability

Combined analysis across the N inputs and seasons revealed that the variance components of general combining ability (GCA) and specific combining ability (SCA) were highly significant (p < 0.05) for all traits (Table 3). Regarding interactions, variance components due to GCA x year and SCA x year were significant for all traits, and variance components associated with GCA x N and SCA x N input and triple GCA and SCA x N input x year interaction were not significant (p > 0.10) only for DTS.

Under both N inputs, the variance components of GCA were significant (p < 0.05) by the likelihood ratio test for almost all agronomic traits, except for GY (Table 4). The N stress affected the genetic divergence among parental hybrids, and the variance components of SCA were not significant (p > 0.10) for almost all traits, except for DTP, under LN, whereas under HN, the variance components of SCA were highly significant (p < 0.05) for all five traits. Concerning the variance components due to interactions, we found that the GCA x year (seasons) interaction was significant (p < 0.05) for DTP, and the SCA x year interaction was significant (p < 0.05) for all traits under LN. However, we found no significant variance component (p > 0.10) due to GCA x year and SCA x year interactions under optimal N conditions.

**Table 4:** Variance components estimates of general (${\hat{\sigma }}_{GCA}^{2}$) and specific combining ability (${\hat{\sigma }}_{SCA}^{2}$), residual variance estimates (${\hat{\sigma }}^{2}$), mean and coefficient of variation (CV%) for five agronomic traits measured in 45 F_1_ crosses evaluated under LH and HN input and over two summer seasons in Brazil.

Parameters	DTP (days)^1/^	DTS (days)	PH (cm)	EH (cm)	GY (kg kg^-1^)
Low N					
${\hat{\sigma }}_{GCA}^{2}$	1.12*^2/^	0.97*	39.20*	46.55*	68,328.11
${\hat{\sigma }}_{SCA}^{2}$	0.86*	0.75	5.32	8.20	151,764.90
${\hat{\sigma }}_{GCAxY}^{2}$	0.29*	0.23	1.93x10^-05^	1.08x10^-05^	721.23
${\hat{\sigma }}_{SCAxY}^{2}$	1.12*	0.97*	39.20*	46.55*	68,328.11
${\hat{\sigma }}^{2}$	2.37	3.09	190.49	106.89	1,600,794.00
Mean	71.17	70.70	211.84	110.55	7,018.04
CV%	2.16	2.25	6.52	9.35	18.03
High N					
${\hat{\sigma }}_{GCA}^{2}$	0.52*	1.06*	25.43*	57.07*	0.12
${\hat{\sigma }}_{SCA}^{2}$	0.32*	0.68*	21.30*	12.96*	720,374.70*
${\hat{\sigma }}_{GCAxY}^{2}$	0.06	0.08	1.31 x10^-05^	8.37x10^-06^	89,716.32
${\hat{\sigma }}_{SCAxY}^{2}$	8.89x10^-07^	2.39x10^-07^	1.31x10^-05^	8.37x10^-06^	128,228.60
${\hat{\sigma }}^{2}$	1.44	2.37	129.78	82.67	1,423,653.00
Mean	67.28	68.88	236.79	128.68	10,509.97
CV%	1.76	2.19	5.04	7.39	14.88

^1/^DTP: days to pollen shedding; DTS: days to silking; PH: plant height; EH: ear height, and GY: grain yield.

^2/^* Significant at 0.05 by the likelihood ratio test.

### GCA and SCA effects

As we found the presence of a GCA x year interaction only for DTS under LN, we estimated the GCA effects for flowering time and plant height traits across seasons at each N input. Even though we found a significant GCA x N input interaction for these traits, we observed a strong coincidence of the GCA effects for plant cycle and plant stature between LN and HN (Table 5). Thus, the parental hybrids BAL188, DKB390, and GNZ7201 were the best under both N inputs since they showed negative GCA effects for DTP, DTS, PH, and EH. Moreover, the hybrid BM3061 showed negative GCA effects for all four traits under HN and for DTS and EH under LN; additionally, BRS1060 showed negative GCA effects for PH and EH under both N inputs and for DTS under HN. The parental hybrids BAL188 and BRS1060 had the lowest GCA effects for cycle and plant stature, respectively, irrespective of N inputs. The other five maize hybrids had positive GCA effects for almost all flowering time and plant stature traits under both N inputs, except BG7049. This parental hybrid had negative GCA effects for PH and EH under LN. Regarding the combined analysis, the hybrids NS70, AG1051, XB8018, GNZ7201, BM3061, and DKB390 presented positive GCA effects for GY across growing seasons and N inputs (data not shown). Additionally, the hybrids BAL188, GNZ7201, DKB390, and BM3061 had negative GCA effects for flowering time and plant stature in the combined analysis.

**Table 5:** Estimates of general combining ability effects of ten parental hybrids for days to pollen shedding (DTP), days to silking (DTS), plant height (PH) and ear height (EH) under LN and HN input across two seasons.

Hybrids	DTP (days)	DTS (days)	PH (cm)	EH (cm)
*Low N*				
AG1051	0.03	0.48	0.86	7.63
BAL188	-2.06	-2.01	-1.70	-6.45
BM3061	0.20	-0.28	2.14	-0.69
BRS1060	0.18	0.17	-10.62	-11.53
DKB390	-0.31	-0.36	-1.01	-0.68
GNZ7201	-1.00	-0.67	-7.48	-5.01
NS70	1.15	0.89	7.03	6.69
SHS4070	0.40	0.55	8.35	6.63
XB8018	0.70	0.58	4.01	6.00
BG7049	0.71	0.66	-1.58	-2.58
		*High N*		
AG1051	0.03	0.44	2.71	8.98
BAL188	-1.44	-1.98	-1.19	-8.26
BM3061	-0.11	-0.17	-0.40	-1.75
BRS1060	0.04	-0.13	-9.57	-13.42
DKB390	-0.28	-0.58	-1.18	-0.81
GNZ7201	-0.52	-0.94	-4.53	-4.35
NS70	0.94	0.96	2.52	3.40
SHS4070	0.40	0.81	6.82	8.87
XB8018	0.43	0.76	2.26	6.41
BG7049	0.51	0.82	2.56	0.92

The SCA was the major component accounting for variation among F_1_ crosses for GY under HN, and the correlation between genotypic values of GY and SCA effects was almost perfect (r = 0.99). Thus, the five top (~10%) F_1_ crosses based on genotypic values of GY also presented the largest values of SCA effects (>600 kg ha^-1^) under HN, with GY values from highest to lowest being: AG1051/GNZ7201 (9,571 kg ha^-1^), BM3061/NS70 (9,527 kg ha^-1^), BM3061/DKB390 (9,333 kg ha^-1^), AG1051/DKB390 (9,197 kg ha^-1^) and GNZ7201/NS70 (9,197 kg ha^-1^; Table 6). All selected F_1_ crosses had at least one parental hybrid that showed negative GCA effects for flowering time and plant stature under HN and across N inputs (combined analysis), and all parents of the five top F_1_ crosses had positive GCA effects for GY across N inputs. We also found that the crossing between hybrids GNZ7201 and DKB390 (-1,334.4 kg ha^-1^) and between AG1051 and NS70 (72.2 kg ha^-1^) presented negative or very small values of SCA effects under HN and across N inputs. Moreover, the parental hybrid BM3061 had high and positive values of SCA effects with the four parental hybrids above under optimal N conditions.

**Table 6:** Estimates of specific combining ability effects above diagonal and predicted genotypic means bellow diagonal for grain yield of 45 maize F_1_ crosses derived from crossing among ten commercial hybrids under high N input across two seasons.

Crosses	AG1051	BAL188	BG7049	BM3061	BRS1060	DKB390	GNZ7201	NS70	SHS4070	XB8018
AG1051	-	38.1	-700.7	554.3	-1,848.1	**800.9**	**1,121.6**	72.1	-156.2	512.1
BAL188	8,393	-	-109.3	-1,785.7	-205.3	-27.5	170.0	434.7	-177.1	171.7
BG7049	7,698	8,135	-	452.2	328.3	-71.3	140.7	551.4	-1,683.2	-625.6
BM3061	8,920	6,683	8,740	-	-1,181.8	**933.6**	480.9	**1,038.5**	-412.0	-111.7
BRS1060	6,588	8,079	8,562	7,194	-	390.7	314.2	76.4	350.1	-225.6
DKB390	**9,196** ^1/^	8,340	8,298	**9,333**	8,718	-	-1,334.4	344.9	175.0	-320.8
GNZ7201	**9,570**	8,572	8,541	8,922	8,680	7,244	-	**637.5**	241.9	53.4
NS70	8,608	8,884	9,027	**9,526**	8,537	8,881	**9,196**	-	-174.6	337.4
SHS4070	8,219	8,106	6,729	7,956	8,592	8,566	8,676	8,369	-	427.9
XB8018	8,922	8,493	7,755	8,295	8,096	8,117	8,572	8,872	8,747	-

^1/^The predicted genotypic values of GY and estimates of SCA effects of the five top (~10%) F_1_ crosses are highlighted in bold.

## Discussion

The evaluation and screening of maize genotypes under low N stress is a prerequisite to develop improved N-use efficiency and high-yielding maize varieties to LN environments. In addition, a deep understanding of the combining ability among commercial hybrids across N inputs is crucial for maize breeders to define breeding strategies to develop new inbred lines and, consequently, modern hybrids more tolerant to LN stress and more efficient in N-use. In our study, we assessed the combining ability among ten commercial hybrids under LN and HN across two seasons. The N stress caused a reduction of means in GY (33.25%) of F_1_ crosses averaged across seasons that fell within the limits of 20–40% set up as the “ideal” level of intensity of N stress for LN tolerance genotype selection [47–49]. The reduction in plant stature under LN stress observed in our study is associated with a decrease in photosynthesis rate, leaf area, and biomass production by plant since LN stress during vegetative development affect carbon assimilation and utilization [50, 51]. In contrast, the increase in flowering time under N stress is mainly related to a delay and reduction in silking emergence due to N deficiency in maize plants during the reproductive stage [52, 53]. Moreover, lower plant stature and larger cycle showed by maize F_1_ crosses under LN stress in our study are in agreement with others results reported in tropical maize evaluated under optimal and LN conditions [18, 19, 54].

In relation to F1 crosses performance under contrasting N inputs, we found the presence of a combining ability (GCA and SCA) x N input interaction for GY, plant stature and DTP, indicating that, for these traits, GCA and SCA effects associated with parental hybrids and crosses, respectively, were not consistent over N inputs. Consequently, the selection of commercial hybrids to be incorporated into a maize breeding program as a germplasm source for developing new inbred lines more low N tolerant and efficient in N-use must be done under each N input. Likewise, [19] emphasized that the screening of maize germplasm based on GCA effects must be conducted separately under each abiotic stress (low N, drought and heat) and under nonstress conditions, mainly for complex traits such as GY and N-use traits.

Many studies have reported differences in the performance of commercial hybrids as parents in crosses for GY in maize under optimal environments [27–30, 55]. However, in our study, the parental hybrids did not differ among them for GY in supplying favorable alleles with additive effects to their progenies, irrespective of N inputs. This result can be attributed to the set of commercial hybrids used in our study since they were previously selected based on their genotypic values for GY and N-use efficiency across contrasting N inputs [17]. Thus, all our parental hybrids have high frequencies of favorable alleles for these traits and genetic potential to be used as sources of alleles for developing new breeding populations and inbred lines with high yielding and N-use efficiency. Then, the selection of the parental hybrids to be incorporated in our maize breeding program can be done based on their GCA effects for flowering time and plant height traits. In agreement with our results, [56] investigated the combining ability among commercial varieties selected for GY under contrasting N inputs and found an absence of a GCA effect for GY and N-use components under LN. In another study, [57] determined that the residual genetic variance for agronomic traits in a maize population improved through several cycles of recurrent selection to drought and low N tolerance and observed the absence of additive and dominance genetic variance for GY and most traits under LN and drought stress.

The introduction of early-maturing and short stature maize germplasm into elite breeding germplasm is crucial for the development of modern hybrids that are more tolerant to LN, drought, stalk lodging and high plant population [19, 58, 59]. In our study, the commercial hybrids showed significant differences in GCA for cycle and plant stature under both N inputs, and their ranks were unchanged between the N inputs (non-crossover type of GCA x N input interaction). Under both N inputs, the parents BAL188, BM3061, DKB390, BR1060 and GNZ7201 had negative GCA effects for almost all flowering time and plant height traits, and they must be used in our breeding program for developing new early-maturing and shorter maize inbred lines with consistent performance across optimum and N stress conditions. Early maturing maize inbred lines and hybrids tend to present greater N-utilization efficiency and grain N concentration and showed more drought tolerant than late maturing, mainly under LN conditions [17, 19, 47, 60]. Finally, it must be pointed out that the early-maturing parents BM3061, DKB390, and GNZ7201 can also be used as sources of favorable alleles for increasing yield across N inputs since they showed positive GCA effects for GY in the combined analysis.

The SCA is the relative performance of a cross that is associated with nonadditive gene action and reflects the differences in the gene frequencies between the parents [61, 62]. In our study, we observed an absence of significance (p > 0.10) of SCA for GY under LN, indicating the nonexistence of allelic complementation among parental hybrids under N stress, whereas nonadditive genes largely modulated the inheritance of the GY in this set of commercial hybrids under HN. Similarly, some studies have reported that SCA was the major component accounting for differences among maize hybrids for GY under HN and that nonadditive gene effects were not important in the inheritance of GY under LN environments [18, 20, 34, 35, 63]. Thus, our results suggested that breeding schemes that capitalize nonadditive gene action (dominance and epistatic effects), such as reciprocal recurrent selection (RRS) and hybridization, must be adopted in our breeding program to improve GY from this set of commercial hybrids targeting optimal N conditions.

The expression of heterosis depends on the presence of some level of dominance among different alleles at a locus and on relative differences in the gene frequency of the two parents [64]. Hence, estimates of SCA effects of traits modulated primarily by nonadditive effects such as GY have been used for clustering germplasm and developing heterotic groups in maize [33, 34, 35, 60, 65]. According to [66], genotypes with positive SCA effects must be allocated into opposite heterotic groups, whereas those with small or negative SCA effects must be allocated into the same heterotic groups. In our study, we intended to develop two synthetic populations by recombining the parental hybrids GNZ7201 and DKB390 (Syn. A), and AG1051 and NS70 (Syn. B) since the crosses between hybrids within each population had negative or very small SCA effects of GY and, beyond that, crosses among parental hybrids from Syn. A with hybrids from Syn. B presented high and positive SCA effects on GY and high yield under HN input. Then, each maize synthetic population will be used as a base population to begin an RRS program among them and, consequently, derive new inbred lines using the synthetic populations and their improved version as a germplasm source.

The RRS method is a highly efficient method for increasing genetic divergence, heterosis and, consequently, crosses between two populations for traits modulated by nonadditive genes [61, 67, 68]. Moreover, the use of synthetic populations derived from commercial hybrids has been recommended for use in RRS programs and has been successful for increasing heterosis and developing genetically diverse inbred lines of maize for Brazilian conditions [24, 26, 29, 37–40, 69]. In addition to synthetic populations, the inbred lines derived from commercial hybrid BM3061 might be crossed and tested with lines developed from both synthetic populations since the crosses between it and four parental hybrids used for developing both synthetics presented high and positive estimates of SCA effects of GY and high yielding under non-N stress conditions. Finally, even though our study was limited to ten parental hybrids, our promising results suggest that commercial hybrids have breeding potential to be used as source germplasm in the development new inbred lines targeting both LN stress and optimal tropical environments.

## Conclusions

We concluded that under both N inputs, the parental hybrids have differences in GCA for flowering time and plant height traits but show similar performance in crossing for GY. Under HN, nonadditive genes largely modulated the inheritance of grain yield in this set of commercial hybrids, whereas there was no SCA effect on grain yield under LN input. The commercial hybrids BAL188, BM3061, GNZ7210, BRS1060 and DKB390 must be selected for developing early- and short-stature breeding populations since they had negative estimates of GCA effects for cycle and plant stature under both N inputs. Additionally, the hybrids GNZ7201 and DKB390 and AG1051 and NS70 must be recombined to develop two synthetic populations to begin a reciprocal recurrent selection program targeting the development of breeding populations and, consequently, genetically diverse inbred lines, mainly for non-N stress environments.
